# Culturally Grounded Scapegoating in Response to Illness and the COVID-19 Pandemic

**DOI:** 10.3389/fpsyg.2021.632641

**Published:** 2021-04-06

**Authors:** Qian Yang, Isaac F. Young, Jialin Wan, Daniel Sullivan

**Affiliations:** ^1^Zhejiang University, Hangzhou, China; ^2^Beloit College, Beloit, WI, United States; ^3^University of Arizona, Tucson, AZ, United States

**Keywords:** scapegoating, medical uncertainty, COVID-19, personal control, China, illness

## Abstract

For years, violence against doctors and healthcare workers has been a growing social issue in China. In a recent series of studies, we provided evidence for a motivated scapegoating account of this violence. Specifically, individuals who feel that the course of their (or their family member's) illness is a threat to their sense of control are more likely to express motivation to aggress against healthcare providers. Drawing on existential theory, we propose that blaming and aggressing against a single individual represents a culturally afforded scapegoating mechanism in China. However, in an era of healthcare crisis (i.e., the global COVID-19 pandemic), it is essential to understand cultural variation in scapegoating in the context of healthcare. We therefore undertook two cross-cultural studies examining how people in the United States and China use different scapegoating responses to re-assert a sense of control during medical uncertainty. One study was conducted prior to the pandemic and allowed us to make an initial validating and exploratory investigation of the constructs of interest. The second study, conducted during the pandemic, was confirmatory and investigated mediation path models. Across the two studies, consistent evidence emerged that, both in response to COVID-related and non-COVID-related illness scenarios, Chinese (relative to U.S.) individuals are more likely to respond by aggressing against an individual doctor, while U.S. (relative to Chinese) individuals are more likely to respond by scapegoating the medical industry/system. Further, Study 2 suggests these culture effects are mediated by differential patterns of primary and secondary control-seeking.

## Introduction

The issue of mistrust between medical patients, on the one hand, and medical providers and professionals on the other, remains a worldwide phenomenon that is arguably growing in recent decades. This issue has taken on an extremely pernicious dimension in the form of violent retaliative acts against doctors and nurses, as well as declining levels of public trust in healthcare institutions more generally. On the international scene, the former problem is especially pronounced in China (The Lancet, 2012, 2014), whereas the latter is especially pronounced in the United States (Wolfensberger and Wrigley, 2019).

With the disastrous global impact of the COVID-19 pandemic, the issue of people's attitudes toward the healthcare system and healthcare workers has become more widely important than ever. Healthcare workers have been subjected to extreme and in many cases unprecedented stressors while dealing with the pandemic (Kröger, 2020), and trust that they will be protected is a key predictor of healthcare worker motivation and well-being during a pandemic (Imai, 2020). It is therefore critical to understand and interrogate how COVID-19 has influenced or failed to influence people's prior trust in and attributions about the healthcare system and healthcare workers. The pandemic also underscores the importance of addressing this imperative from a cross-cultural perspective. Of particular importance for the present project is the fact that, despite the apparent origination of the COVID-19 outbreak in China, the spread and consequences of the virus have been more severe to date in the United States relative to China (Hua, 2020; Lo and Shi, 2020).

In the current project, we hope to shed light on how the pandemic may have exacerbated cross-cultural variation in attitudes toward healthcare as a function of medical uncertainty. We present the first systematic evidence to date concerning differences in how people in China and the United States respond to the anxiety of medical uncertainty with compensatory psychological defense mechanisms. We adopt a cultural perspective on scapegoating (Sullivan et al., 2014), which suggests that, universally, people may react to the anxious uncertainty of loss of personal control by scapegoating—disproportionately blaming and/or aggressing against—particular viable targets. However, the viability of a target is in large part determined by cultural factors. Specifically, we expected that whereas targeted aggression against specific healthcare workers may be a culturally afforded scapegoating mechanism in China, people in the United States may be comparatively more likely to blame the healthcare system as a whole in the face of medical uncertainty. We further expected these differences in culturally afforded scapegoating to be mediated by different patterns of control-seeking in the different cultural contexts. We tested these ideas in an initial exploratory study conducted prior to the outbreak of the COVID-19 pandemic (Study 1), and then performed a confirmatory study investigating the robustness of these relationships during the pandemic (Study 2).

## Scapegoating in the Face of Medical Uncertainty

The current research examines a specific psychological mechanism that we propose contributes to violence against doctors and nurses in China, and to healthcare system distrust in the United States: namely, lack of perceived personal control on the part of patients and their relatives in situations of heightened medical uncertainty. Our present model draws on current research and theory regarding the psychological process of *scapegoating* as a control maintenance mechanism (Sullivan et al., 2014). Studies show that when people are threatened by perceptions of uncontrollability in their lives, they evince an increased tendency to attribute blame and power to enemy individuals, groups, and organizations who may be scapegoated (Rothschild et al., 2012). Cognitively and motivationally, it is reassuring to see evil in the world not as due to random, unpredictable forces, but rather as stemming from focal individuals who can be controlled and on whom one can exact retribution, or from organizations and institutions that can be politically or economically held accountable.

Undergoing experiences of illness, whether one's own or that of loved ones, can be a major threat to perceived personal control. Thus, it stands to reason that in situations of medical uncertainty (e.g., a chaotic disease course, or contracting COVID-19 in the midst of a global pandemic), people will be motivated to scapegoat particular targets to which blame for the illness and its effects may be attributed^1^. However, we crucially propose that the cultural context in which individuals are immersed will influence both (a) the exact nature of the control-seeking motive they are seeking to satisfy in the uncertain situation, and (b) the nature of the target that will be afforded as most viable for blame and attendant aggression or distrust.

## Cultural Pathways: Control-Seeking and Trust in China and the United States

Our research can be understood in terms of a *cultural pathways* approach, which suggests that relatively universal psychological processes—such as the motive to maintain perceived control over one's health, and to make attributions when that control is threatened—are shaped by particular cultural imperatives and affordances (Kitayama et al., 2010). We assert that different cultural patterns of control-seeking and trust in the United States and China are important in this regard. First, we emphasize the distinction between *primary* and *secondary* control-seeking. As originally defined by Rothbaum et al. (1982), primary control-seeking refers to attempts to influence one's environment to suit the desires of the self, and is a predominant cultural imperative in more historically independent settings such as the United States. On the other hand, secondary control-seeking refers to a set of strategies for adapting the self to fit environmental requirements, and is a more common imperative in historically interdependent settings such as China (Rothbaum et al., 1982). In particular, in the healthcare context, a form of secondary control-seeking labeled *vicarious control*—putting trust in powerful others and authority figures to control the self's outcomes (Rothbaum et al., 1982)—is of special relevance, given the fact that patients are placing their well-being in the hands of healthcare professionals.

It is also critical to take into account divergent cultural patterns of trust when it comes to understanding how lay people relate to the healthcare system and workers, particularly in context of medical uncertainty. In this regard, we must distinguish between different levels and types of trust, given that people's interactions with healthcare workers are of a local and interpersonal (albeit professional) nature, whereas their beliefs about the broader healthcare system represent a form of institutional or governmental trust. Generally, recent research on the cultural psychology of trust (Liu et al., 2018; Zhang et al., 2019) suggests that people in the United States and in China have relatively different patterns of trust at the interpersonal and institutional/political levels. To summarize this research cursorily, people in the United States have relatively high levels of interpersonal, but relatively low levels of institutional trust; whereas people in China tend to have more comparable levels of trust across persons and institutions. Indeed, Chinese people evidence a relatively unique, “top-down” structure of trust reflecting the centralized nature of the Chinese government, such that people tend to have high levels of trust in the overall governmental system, but lower levels of trust in local representatives of institutions (Zhang et al., 2019).

In China, research suggests that traditionally people are oriented toward more passive forms of coping with stressors (such as illness) by adjusting the self to better fit the environment, or to restore a kind of imbalance between the person/body and the environment (Cheng et al., 2010; Unschuld, 2018). Thus, people in contemporary China may be oriented toward seeking secondary control when it comes to their health, and in particular toward vicarious control—for instance, they may wish to place their trust in physicians. By contrast, we expect people in the United States (particularly from higher SES backgrounds) to have more of a primary control-seeking orientation toward the health domain. People in the United States may be especially likely to view themselves as “consumers” of healthcare services, and expect that their needs for autonomy and full information will be honored when they consult with healthcare experts. For example, Alden et al. (2015) found that among U.S. (but not Japanese) participants, independence values were related to the desire for shared decision-making in medical situations.

Surprisingly, cultural psychological research on trust has generally not assessed people's level of trust specifically in the healthcare domain (Liu et al., 2018; Zhang et al., 2019). But given the broader patterns of trust described above, it is reasonable to assume that in China, people may have relative trust in the national healthcare system overall, but less trust in local representatives of that system (healthcare workers); whereas in the United States, this relationship may take the opposite form. We now consider more applied research on developments in doctor-patient relationships and healthcare system trust in these two countries, applying the theoretical constructs of culturally-patterned scapegoating, control-seeking, and trust to illuminate these developments.

## Aggression Against Healthcare Workers in China

Prior to the COVID-19 pandemic, levels of aggression, and violence against healthcare professionals in China in recent years had nearly reached the state of a public emergency. These acts have a clear negative impact on the mental well-being of professionals in China. In a sample of nearly 2,500 medical providers from the Fujian and Henan Provinces, 50% reported at least one incident of patient-inflicted violence over the previous year, and experience of violence was a significant negative predictor of quality of life even controlling for other relevant factors (Wu et al., 2014). Indeed, many medical professionals in China now report regretting their choice of career, leading some to anticipate an impending crisis in the health services.

Explanations for this phenomenon in China typically focus on social structural and economic causes. The troubled transition to the commodification of medical services in China since 1980 has led to widespread issues of mismatched expectations and insufficient funds and insurance for healthcare on the part of the public (Hesketh et al., 2012; The Lancet, 2014). From the side of medical providers, overwork and underpayment combines with a problematic incentive structure to generate over-prescription and a lack of face-time with patients (He, 2014).

While such explanations and corresponding intervention recommendations are clearly important, we propose that it is also crucial to understand the psychological mechanism(s) underlying the rise in violence against medical professionals.

Two assumptions from the preceding section may explain the cultural pathway to scapegoating of these professionals in the Chinese context. First, people in China are motivated to seek secondary, and particularly vicarious, forms of control in the healthcare context; and second, people in China have relatively high trust in central institutions but relatively low trust in local institutional representatives. This combination of factors suggests that, in the face of medical uncertainty or frustration, Chinese individuals will be relatively likely to aggress against the healthcare workers in whom they had hoped to place their trust, but who appear to have failed them. Beyond testing this empirical account, it is also important to understand if these same factors persist under the recent conditions of the COVID-19 pandemic.

## Distrust of the Healthcare System in the United States

Attitudes toward healthcare on the part of the public are also becoming increasingly negative in the United States in recent decades. This shift has happened less on the terrain of attitudes toward and aggression against individual healthcare workers, and more on the level of institutional trust toward the healthcare system, which has declined in the United States over the past half-century (Wolfensberger and Wrigley, 2019). For example, a variety of studies have documented variation in healthcare system trust as an important determinant of use of medical care and health-relevant outcomes in the United States (Shea et al., 2008). It is important to acknowledge that at least some data suggest these general declines in institutional trust are independent of people's interpersonal trust in their own physicians (Hall, 2005).

A number of sociological explanations have been proposed for this decline in healthcare system trust. Prominent among these is the general commercialization and privatization of healthcare in the United States, which prompts individuals to suspect the healthcare system and “Big Pharma” of exploiting people's health problems for profit (Wolfensberger and Wrigley, 2019). Healthcare issues have also become heavily politicized in the United States in recent years, with global trends toward political polarization finding one lightning rod in debates around the Affordable Care Act (Béland et al., 2016). The issue of public trust in the healthcare system, professionals, and epidemiologists clearly played a role in the U.S. national response to the COVID-19 pandemic. To be specific, high public levels of distrust in medical professionals, which could be strategically stoked by the Trump Administration, almost certainly contributed to this nation's relatively costly and ineffective public health response (Lo and Shi, 2020).

As in the case of the rise of aggression against healthcare workers in China, we believe it is important to understand patterns in healthcare system (dis-)trust in the United States from a psychological vantage. The cultural pathway to scapegoating of the healthcare system in the United States may be explained by our assumptions about control-seeking and the cultural psychology of trust. Many people in the United States may find their motives for primary control-seeking frustrated in the health domain, particularly in light of rising costs of medical care, lack of insurance for many residents, and the current seriousness of the COVID-19 outbreak (Shi and Stevens, 2010; Burton et al., 2020). But given that U.S. residents typically show a combination of low governmental/institutional and high interpersonal trust, they would likely respond to these threats not primarily by aggressing against their local healthcare providers, but rather with increasing distrust of the healthcare system. This novel account has not yet been tested due to a lack of attention to healthcare trust in the cultural psychology literature.

In sum, our framework makes the following predictions:

*Hypothesis 1: People in China (vs. the United States) will have a greater tendency to aggress against specific healthcare workers in situations of medical uncertainty; whereas people in the United States (vs. China) will show greater tendencies to distrust the healthcare system as a whole*.

*Hypothesis 2: These culture-level differences in scapegoating mechanisms will be partially mediated by different patterns of control-seeking, such that primary control-seeking will partially explain U.S. individuals' greater health system distrust, and secondary control-seeking will partially explain Chinese individuals' greater aggression against doctors*.

## Prior Research Supporting the Framework in China

Some prior evidence supports the first half of our framework, namely, that threats to control in the medical context are associated with greater aggression against doctors among Chinese participants. Yang et al. (under review) demonstrated that Chinese people tend to blame doctors for the outcomes of uncertain medical scenarios to a greater extent when they dispositionally lack control. An additional study examined whether a situational threat to control would make participants more likely to blame doctors. Yang et al. (under review) asked participants to read scenarios about a patient's experience in the hospital. They manipulated whether the disease course was chaotic (and thus control-threatening) or not, and whether the patient's condition improves or worsens at the end of the narrative. They predicted that participants would attribute more responsibility to doctors when the patient's condition turned worse and the disease course was chaotic; i.e., doctor blaming would serve the psychological need to make sense of uncontrollable suffering by scapegoating a focal human agent.

Importantly, this study recruited participants from both China and the United States. Consistent with the current model, among Chinese participants, there was a strong interaction effect such that, when a patient's condition worsened in a scenario, attribution of blame to doctors was especially high when the disease course was chaotic. While a similar effect was observed among U.S. participants, it was much less pronounced, and overall U.S. participants tended to attribute more responsibility to doctors when the hypothetical course of a patient's illness was positive (a main effect not observed in Chinese participants).

These suggestive prior studies leave questions unanswered when it comes to our theoretical framework. Specifically, they failed to distinguish between motives for primary and secondary control, they did not assess healthcare system distrust, and—most important in the present context—they were conducted prior to the COVID-19 pandemic, and so did not examine these important processes in light of this historical event. To address these issues, we conducted two surveys comparing Chinese and U.S. samples. Study 1 was conducted prior to the COVID-19 pandemic, and represented an exploratory first attempt to test Hypothesis 1 of our framework, as well as the suitability of different measures of our variables for testing the model. After the pandemic began, we carried out Study 2 as a confirmatory test of Hypotheses 1 and 2. We did not have a strong *a priori* rationale to expect that the experience of COVID-19 would change the processes specified by our theoretical account; if anything, we expected the strong threat to control posed by the pandemic to exacerbate these culturally specific processes.

## Study 1

### Method

Participants first responded to a series of measures localized to the healthcare context, including health-specific LOC (Wallston et al., 1978), health system distrust (Shea et al., 2008), and fatalism in personal health (Shen et al., 2009)^2^. Participants next responded to general measures of perceived control, specifically the personal mastery and perceived constraint subscales developed by Michinov (2005). Finally, participants responded to a series of vignettes that described uncertainty-inducing healthcare experiences (e.g., waiting for days in a hospital for an operation, being prescribed an expensive medication, and being sent home with a different diagnosis the day before a scheduled surgery). They were asked about their level of frustration, and their desire to aggress against the healthcare provider in each scenario.

#### Participants

To assess culturally shaped responses to healthcare, Study 1 administered measures to Chinese and U.S. participants. In both the U.S. and China, data were collected from online participant recruitment platforms (Amazon Mechanical Turk and Zhubajie, respectively). Data collection initially resulted in a total of 692 responses (363 U.S., 329 Chinese), but the elimination of participants who failed to correctly respond to attention checks resulted in final samples of 317 American and 329 Chinese respondents. Participants were compensated with $1.50 in the U.S. and 10RMB in China for their time and effort. Though the samples are roughly comparable in terms of being drawn from online participant populations, there were demographic differences in terms of age [*M*_U.S._ = 35.72, *SD*_U.S._ = 11.73; *M*_China_ = 31.46, *SD*_China_ = 7.47; *t*_(644)_ = 5.53, *p* < 0.001] and gender (for U.S., 59% male and 40% female; for China, 41% male and 59% female; $\chi_{\left(2\right)}^{2}$ = 23.74, *p* < 0.001)^3^.

#### Materials

When possible, existing and validated translations of measures were used for the Chinese participants. When this was not possible, a back translation process was utilized, in which a native Chinese speaker not involved with the research process translated into English the items that had been translated by the researchers, and any discrepancies with respect to the original English-language items were resolved.

##### Healthcare-Specific Control Measures

Participants first completed measures assessing perceptions of control and control-seeking tendencies specifically in the context of healthcare and personal health. The first of these was the health-specific LOC measure (Form A; Wallston et al., 1978), to which participants responded on a 6-point scale (higher scores indicating greater agreement with a target statement). This 18-item measure breaks into 3 subscales. Internal Health LOC (HLOC; α = 0.65) consists of items such as “I am in control of my health.” Powerful Others HLOC (α = 0.59) consists of items such as “Health professionals control my health.” Chance HLOC (α = 0.66) consists of items such as “Most things that affect my health happen to me by accident.” Participants also completed a measure of health-specific fatalism, the “Predetermination” subscale from the Shen et al. (2009) measure, to which participants responded on a 5-point scale (higher scores indicating greater agreement with a target statement). This 10-item scale (α = 0.88) consists of items such as “My health is determined by fate.”

##### Global Control Measures

Participants also completed Michinov's (2005) measure of general perceived control, to which participants responded on a 5-point scale (higher scores indicating greater agreement with a target statement). The 12-item measure is broken down into 2 subscales. The Personal Mastery scale (4 items; α = 0.76) consists of items such as “What happens to me in the future mostly depends on me.” The Perceived Constraint scale (8 items; α = 0.87) consists of items such as “What happens in my life is often beyond my control.”

##### Outcome Measures

Participants also completed measures of our primary theorized outcomes of interest (note that this is an initial cross-sectional and exploratory investigation). The first was Health System Distrust, assessed with the scale developed by Shea et al. (2008), to which participants responded on a 5-point scale (higher scores indicating greater agreement with a target statement, and thus greater *distrust* of the health system). This 9-item measure (α = 0.80) consists of items such as “The Health Care System lies to make money.”

The second outcome measure was aggression against doctors. This measure was validated in prior research in China (Yang et al., under review). Participants responded to 3 vignettes that described uncertainty-inducing healthcare experiences (e.g., waiting for days in a hospital for an operation, being prescribed an expensive medication). For each scenario, participants responded to 2 items. The first indexed *frustration* with the scenario and the healthcare provider: “To what extent are you frustrated with the doctor's behavior?” (*1* = *no frustration at all*; *5* = *a lot of frustration*). The second indexed the primary theorized outcome of *aggression against doctors*: “To what extent do you have the urge to hit the doctor?” (*1* = *have no intention at all*; *5* = *a very strong intention*). We created composite indices by averaging responses to each item type across the 3 scenarios (for frustration, α = 0.57; for aggression against doctors, α = 0.75).

### Results

#### Culture Mean-Level Differences

The current study was conducted in an exploratory fashion. Nevertheless, we hypothesized that there would be certain mean-level differences between the two cultural groups. Specifically, we expected that U.S. participants would score relatively higher on measures of primary control-seeking and Chinese participants would score relatively higher on measures of secondary control-seeking. We also expected that whereas Chinese participants would score relatively higher on aggression against doctors, U.S. participants would score relatively higher on health system distrust. All descriptive statistics are presented in Table 1.

**Table 1:** Within-country zero-order correlations and descriptives (Study 1).

	**Mean (SD) Chinese Sample**	**1**	**2**	**3**	**4**	**5**	**6**	**7**	**8**	**9**	**Mean (SD) U.S. Sample**
1. Internal HLOC	4.12 (0.75)	–	0.57^**^	−0.10	−0.45^**^	−0.31^**^	−0.43^**^	−0.14^*^	0.01	0.03	4.47 (0.82)
2. Perceived mastery	3.97 (0.56)	0.26^**^	–	−0.07	−0.32^**^	−0.17^**^	−0.58^**^	−0.20^**^	−0.06	−0.08	3.84 (0.81)
3. Others HLOC	3.79 (0.82)	0.25^**^	0.28^**^	–	0.39^**^	0.32^**^	0.22^**^	−0.16^**^	−0.06	0.24^**^	3.00 (1.00)
4. Chance HLOC	2.70 (0.67)	−0.05	−0.14^*^	0.09	–	0.68^**^	0.57^**^	0.25^**^	0.09	0.37^**^	2.75 (1.00)
5. Fatalism	2.41 (0.62)	−0.13^*^	−0.22^**^	−0.11^*^	0.46^**^	–	0.48^**^	0.17^**^	0.10	0.34^**^	2.19 (0.91)
6. Perceived constraint	2.68 (0.67)	−0.18^**^	−0.41^**^	−0.16^**^	0.33^**^	0.38^**^	–	0.30^**^	0.19^**^	0.26^**^	2.34 (0.92)
7. Health system distrust	2.81 (0.62)	−0.20^**^	−0.37^**^	−0.29^**^	0.20^**^	0.29^**^	0.33^**^	–	0.27^**^	0.16^**^	3.33 (0.84)
8. Scenario frustration	2.67 (0.95)	−0.00	−0.20^**^	−0.09	0.09	0.15^**^	0.16^**^	0.19^**^	–	0.36^**^	2.58 (0.97)
9. Aggression against doctors	1.82 (0.89)	0.04	−0.16^**^	−0.02	0.13^*^	0.16^**^	0.21^**^	0.21^**^	0.76^**^	–	1.35 (0.73)

*Results for the Chinese sample are reported below, the U.S. sample above the diagonal*.

* *p < 0.05*,

** *p < 0.01*.

##### Primary Control-Seeking

We had one health-specific measure (Internal HLOC) and one global measure (Personal Mastery) of primary control-seeking. As expected, U.S. participants scored higher on Internal HLOC, *t*_(644)_ = 5.65, *p* < 0.001, *d* = 0.45. However, contrary to expectations, Chinese participants scored higher on Personal Mastery, *t*_(644)_ = −2.33, *p* = 0.02, *d* = 0.19.

##### Secondary Control-Seeking

We had three health-specific measures (Powerful Others and Chance HLOC; Fatalism) and one global measure (Perceived Constraint) of secondary control-seeking. As expected, Chinese participants scored higher on Powerful Others HLOC, *t*_(644)_ = −11.01, *p* < 0.001, *d* = 0.87, Fatalism, *t*_(644)_ = −3.74, *p* < 0.001, *d* = 0.30, and Perceived Constraint, *t*_(644)_ = −5.38, *p* < 0.001, *d* = 0.42. However, contrary to expectations, there was no observed culture difference on Chance HLOC, *t*_(644)_ = 0.71, *p* = 0.48.

##### Outcome Measures

As expected, U.S. participants scored higher overall in health system distrust, *t*_(644)_ = 8.86, *p* < 0.001, *d* = 0.70, while Chinese participants scored higher in aggression against doctors, *t*_(644)_ = −7.41, *p* < 0.001, *d* = 0.58. Interestingly, participants from the two cultures did not differ in their expressed level of frustration at the medical uncertainty scenarios, *t*_(644)_ = −1.21, *p* = 0.23.

#### Patterns of Association

This exploratory study had two primary purposes. The first was to test our expectations concerning culture mean-level differences. The second was to examine patterns of association among the variables, in order to determine which operationalizations of primary and secondary control-seeking might be most effective to use in a subsequent confirmatory study testing our multiple mediator path model. To reiterate, our guiding model suggests that relative tendencies toward health system distrust in the United States should be driven by primary control-seeking, whereas relative tendencies toward aggression against doctors in China should be driven by secondary control-seeking.

Within-country correlations are presented in Table 1; however, we examined associations across the entire dataset in order to determine which variables would be most important to include in a subsequent confirmatory study (Table 2). We eliminated Chance HLOC from our deliberations, because there was no culture mean-level difference on this variable, suggesting it would be unlikely to be a useful indicator for our model in a subsequent study.

**Table 2:** Correlations of primary interest for the whole dataset (Study 1).

	**1**	**2**	**3**	**4**	**5**	**6**	**7**
1. Health system distrust	—						
2. Aggression against doctors	0.07	—					
3. Internal HLOC	−0.08^*^	−0.03	—				
4. Personal mastery	−0.27^**^	−0.05	0.45^**^	—			
5. Powerful others HLOC	−0.31^**^	0.20^**^	−0.04	0.09^*^	—		
6. Fatalism	0.15^**^	0.27^**^	−0.26^**^	−0.17^**^	0.20^**^	—	
7. Perceived constraint	0.22^*^	0.27^**^	−0.36^**^	−0.49^**^	0.15^**^	0.46^**^	—

N = 646

* *p < 0.05*.

** *p < 0.01*.

We noted that our measure of health system distrust was related to our measures of primary control-seeking. However, in both cases these relationships were *negative*, rather than positive as our theoretical model would suggest. In other words, participants who scored higher in Internal HLOC or Personal Mastery reported *less* health system distrust.

We noted that our measure of aggression against doctors was not related to our primary control-seeking measures, and instead consistently positively related to our secondary control-seeking measures, as our model would suggest. However, we additionally noted that among the secondary control-seeking measures, Powerful Others HLOC was best able to discriminate between the outcome measures, because it was negatively related to health system distrust, but positively related to aggression against doctors. On the other hand, the other secondary control-seeking measures (Fatalism and Perceived Constraint) seemed to be associated with general negativity toward healthcare (i.e., higher health system distrust *and* aggression against doctors).

### Discussion

Our initial exploratory study yielded several preliminary conclusions that helped shape our subsequent confirmatory study designed to test our multiple mediator path model. First, mean-level comparisons generally supported our expectations for cross-cultural differences: U.S. participants scored higher on health system distrust, whereas Chinese participants scored higher on aggression against doctors. In addition, Chinese participants scored higher on our secondary control-seeking measures. Given that participants from the two countries scored similarly in the level of frustration they expressed at the medical uncertainty scenarios, this provides initial support for our guiding framework, which suggests that people in China and the United States have relative tendencies to resolve tensions in the healthcare domain using different culturally afforded defenses.

Given cross-cultural differences in these important applied phenomena (aggression against doctors and healthcare system distrust), a critical task is to determine the cultural pathways that afford these divergent responses across national settings. Examination of the mean-level differences and overall patterns of association yielded additional useful information. We were particularly interested in distinguishing between our different measures of primary and secondary control-seeking to prepare our subsequent confirmatory study. When it came to primary control-seeking, the measures did not perform in expected ways for two apparent reasons. First, contrary to expectations and the prior literature, Chinese (relative to U.S.) participants scored higher on the Personal Mastery measure. Second, these measures were associated with health system distrust, but in a negative direction.

In hindsight, these patterns were not surprising given the important distinction between *presence of control* and *desire for control*, which has been noted in prior literature, but to which we paid insufficient attention in designing Study 1 (Burger and Cooper, 1979). The Study 1 results suggest that if a patient already has their needs for primary control satisfied, they do not need to invoke culturally afforded defenses in connection with the healthcare system. And indeed, our theoretical account only suggests that desire for, rather than presence of, primary control should be associated with scapegoating defenses. This indicated to us that we should select a new measure of primary control-seeking for Study 2, specifically a measure that indicated not presence of but desire for primary control in the medical domain. If we could operationalize participants' desire for a primary control that they currently lack, this might be positively associated with use of health system distrust as a defense mechanism, at least among U.S. participants.

When it came to secondary control-seeking, the measure of Powerful Others HLOC seemed most promising for a subsequent study. Of the secondary-control seeking measures, this was the only one to show a culture mean-level difference with a large effect size (in the expected direction). In addition, this measure distinguished well between our two outcomes, in that it was negatively associated with health system distrust, but positively associated with aggression against doctors. This suggests that specifically seeking secondary control in the health domain by yielding power to others may be associated with the culturally afforded defense of violence against healthcare workers, at least among Chinese participants. These findings fit with our theoretical account given the importance of vicarious control as a specific form of secondary control-seeking (Rothbaum et al., 1982) in the medical domain (e.g., Goodyear-Smith and Buetow, 2001).

## Study 2

We had two primary goals for Study 2. First, we planned to replicate and extend our exploratory Study 1 findings in light of our guiding hypotheses. Hypothesis 1 was supported in Study 1, but we wanted to confirm this pattern in a second sample. In addition, we wanted to test Hypothesis 2 using a confirmatory approach and applying multiple-mediator path models. We planned to use the information from Study 1 regarding which operationalizations were most effective and consistent with our theoretical framework to update the materials for Study 2. Specifically, we observed that Powerful Others HLOC was a promising operationalization of vicarious control as a relevant form of secondary control-seeking in the healthcare context; and we also felt the need to develop a new measure of primary control-seeking that would indicate *desire for*, rather than presence of, primary personal control in the healthcare context.

But second, the COVID-19 pandemic occurred before we were able to follow up on our Study 1 results. Due to the obvious importance of the pandemic for people's experiences of medical uncertainty, we additionally modified the Study 1 materials to include vignettes pertaining to the COVID-19 situation. Given the historic moment, an additional goal of Study 2 became determining whether the Study 1 findings, and our original hypothesized relationships, would be observable during the pandemic. We had no strong reason to believe *a priori* that the basic pattern of results would change, and therefore retained our original hypotheses.

### Method

Data were collected at the beginning of May, 2020. Similar to the procedure of Study 1, participants first responded to a series of vignettes that described uncertainty-inducing healthcare experiences. However, Study 2 also included scenarios related to the COVID-19 pandemic. Following the healthcare vignettes, participants responded to measures of primary control-seeking (shared decision-making), health system distrust, secondary control-seeking, and positive cognitive reframing. For descriptive statistics and zero-order correlations for all the variables reported below, see Table 3. Finally, because the threat of COVID-19 may have been experienced by participants as more distal or proximal depending on whether they lived in an area that was heavily impacted by the virus, a single item was included to assess whether participants had lived or stayed in a region impacted by COVID-19.

**Table 3:** Within–country zero-order correlations and descriptives (Study 2).

	**Mean (SD) Chinese Sample**	**1**	**2**	**3**	**4**	**5**	**6**	**7**	**8**	**Mean (SD) U.S. Sample**
1. Primary control-seeking^*^	3.64 (0.66)	–	−0.21^**^	0.17^**^	0.14^**^	0.08	0.21	−0.01	0.12	4.11 (0.79)
2. Secondary control-seeking^*^	4.69 (0.86)	−0.19^**^	–	−0.12^*^	0.28^**^	0.04	0.32^**^	0.02	0.36	3.88 (1.16)
3. Health system distrust^*^	2.84 (0.86)	0.36^**^	−0.30^**^	–	−0.24^**^	0.26^**^	0.18^**^	0.19^**^	0.13^*^	3.81 (1.11)
4. Positive cognitive reframing^*^	5.48 (0.87)	−0.06	0.28^**^	−0.25^**^	–	−0.08	0.18^**^	−0.08	0.16^**^	4.43 (1.59)
5. General scenario frustration	2.42 (0.91)	0.19^**^	−0.16^**^	0.27^**^	−0.06 ^**^	–	0.39^**^	0.42^**^	0.27^**^	2.54 (0.91)
6. General aggression against doctors^*^	1.63 (0.78)	0.14^**^	−0.07	0.23^**^	0.0.02	0.64^**^	–	0.06	0.58^**^	1.40 (0.76)
7. COVID frustration^*^	2.95 (1.03)	0.14^**^	−0.05	0.21^**^	0.02^**^	45^**^	0.36^**^	–	0.45^**^	3.30 (1.04)
8. COVID aggression against doctors^*^	2.19 (1.05)	0.15^**^	0.00	0.22^**^	0.03	0.42^**^	0.55^**^	0.78^**^	–	1.83 (1.01)

*Results for the Chinese sample are reported below, the U.S. sample above the diagonal*.

* *Indicates significant mean-level differences between countries at p < 0.001. For correlations*,

** *p < 0.01*.

#### Participants

To assess culturally shaped responses to healthcare in the era of the COVID-19 pandemic, Study 2 administered several measures to Chinese and the American participants. In both the U.S. and China, data were collected from online participant recruitment platforms (Amazon Mechanical Turk and Zhubajie, respectively). *Post-hoc* power analyses of primary dependent variables' from Study 1 suggest that study's sample size resulted in sufficient power (power = 1.00). Based on the Cohen's *d*s from Study 1 for health system distrust (0.70) and aggression toward doctors (0.58), a priori power analyses suggest that a sample size between 68 and 96 is necessary to achieve power of 0.80 for detecting these differences again. However, in order to examine mediational pathways by which nation-level differences, we sought to maximize the sample size within constraints of available resources. Data collection initially resulted in a total of 1,251 responses (653 U.S., 562 Chinese), but the elimination of participants who failed to correctly respond to attention checks resulted in final samples of 370 U.S. and 551 Chinese respondents. Participants were compensated with $1.5 in the United States and 10RMB in China. As in Study 1, age was higher overall and more varied in the United States (*M*_U.S._ = 40.42, *SD*_U.S._ = 12.42; *M*_China_ = 30.25, *SD*_China_ = 8.45; *t*_(919)_ = 14.92, *p* < 0.001). Gender differences were similar to those observed in Study 1 as well, although not as pronounced (for U.S., 58.1% male and 40.5% female; for China, 46.5% male and 53.5% female; $\chi_{\left(1\right)}^{2}$ = 13.61, *p* < 0.001)^4^. In addition, an examination of the item probing whether participants lived in an area impacted by the virus revealed that significantly more American (compared to Chinese) participants reported living in a virus-affected area (for U.S., 62.8% lived in unaffected areas and 37.2% lived in affected areas; for China, 85.7% lived in unaffected areas and 14.3% lived in affected areas; $\chi_{\left(1\right)}^{2}$ = 64.30, *p* < 0.001).

#### Materials

##### Healthcare Uncertainty Vignettes

Participants first reported their frustration and desire to aggress in response to the series of scenarios reported in Study 1. Then, participants read and responded to scenarios that related to potential healthcare situations involving the COVID-19 pandemic. For example, in one vignette, participants read about the following scenario: “Imagine your grandfather has had a high fever for 5 days at this time. After going to the hospital for a blood test and CT test, he was highly suspected of having new coronavirus pneumonia. Since there were no vacant ward beds in the hospital, the doctor prescribed medicine and let the patient go home for isolation.” Similar to the general vignettes, participants reported their predicted frustration and desire to aggress against the doctor based on each scenario. Responses were provided on 5-point Likert scales.

##### Primary Control-Seeking

To assess participants' desire for personal control in their healthcare, participants responded to a modified version of the Desirability for Control scale (Gebhardt and Brosschot, 2002). This scale includes three subscales, all of which were modified to reflect decision-making in healthcare contexts, including desire for leadership (e.g., “I enjoy participating in medical decisions, because I want to have as much of a say in treatment options as possible”), willingness to relinquish control (reverse coded, e.g., “I wish I could push the medical decisions off on my doctor”), and desire for determining one's own life (e.g., “I enjoy making my own decisions”; across all subscales, a = 0.82).

##### Secondary Control-Seeking

The full health-specific locus of control scale (Wallston et al., 1978) was again included, but based on the exploratory Study 1 results and our theoretical framework the subscale measuring trust in powerful others (vicarious control-seeking) was the focus for the present study (a = 0.77).

##### Health System Distrust

Health system distrust was assessed with the same measure used in Study 1 (a = 0.89).

##### Positive Cognitive Reframing

As an exploratory measure, a measure of positive cognitive reframing was included to assess the degree to which individuals positively reinterpret their healthcare experience. We included this measure because recent evidence suggests that people in China have shown more positive forms of coping with the COVID-19 pandemic compared to U.S. residents (Ji et al., 2020). Accordingly, while we did not formulate new hypotheses for Study 2, we wanted to explore the possibility that Chinese residents might show more positive coping in the COVID-19 context, rather than aggression against doctors. The 4-item measure was taken from the COPE inventory (a = 0.84; Carver et al., 1989).

### Results

#### Invariance Analyses of Primary Outcomes

In order to determine the degree of factor structure similarity between the U.S. and China for the primary dependent variables, invariance analyses of health system distrust and aggression toward doctors (both the general and COVID-specific scenarios) were conducted. A confirmatory factor analysis (CFA) model was specified in which health system distrust and aggression toward doctors were treated as latent factors with their respective items serving as the indicators. By adding constraints to these models, we can determine whether the items are capturing the same underlying construct (configural invariance, established through a multigroup CFA), whether participants in both nations are similarly responding to the items (metric invariance, established by constraining factor loadings to be equivalent between groups), and whether the means are comparable (scalar invariance, established by constraining intercepts to be equivalent between groups). These analyses were conducted in the R software package and utilized weighted least squares estimators and robust fit indices. The acceptability of different levels of invariance can be determined by examining changes in fit statistics. While chi-square changes can be overly sensitive, CFI and Gamma-hat can be examined for changes to determine whether each consecutive model should be rejected, with changes of <0.01 indicating that the more constrained model is acceptable (Milfont and Fischer, 2010). Fit statistics for these CFAs are presented in Table 4.

**Table 4:** Fit statistics for invariance models (Study 2).

**Model**	***X*^**2**^ (scaled)**	**Robust CFI**	**Robust TLI**	**Gamma-hat**	**Robust RMSEA [90% CI]**	**SRMR**
**Health system distrust and aggression against doctors (general)**						
Configural	684.27	0.947	0.934	0.965	0.073 [0.068, 0.079]	0.075
Metric	640.33	0.941	0.933	0.959	0.074 [0.068, 0.080]	0.081
Scalar	866.42	0.916	0.912	0.938	0.085 [0.080, 0.090)	0.091
**Health system distrust and aggression against doctors (COVID)**						
Configural	706.64	0.944	0.93	0.959	0.077 [0.072, 0.083]	0.076
Metric	663.758	0.939	0.931	0.954	0.077 [0.071, 0.083]	0.081
Scalar	888.519	0.914	0.91	0.934	0.088 [0.082, 0.093]	0.092

In the case of both sets of models—one examining health system distrust and aggression in the general healthcare scenarios and the other examining health system distrust and aggression in the COVID-19 specific scenarios—the configural metric models had acceptable fit and all factor loadings were significant (*p* < 0.001). Further, the constraints added to the metric models did not lead to a substantial decrease in the model fit (i.e., Δ CFI and Δ Gamma-hat <0.01). In both cases, the implementation of additional constraints in the scalar models resulted in worse model fit (though still acceptable with more liberal fit cutoffs; e.g., RMSEA <0.10). This is not surprising as scalar invariance is a high psychometric standard for between-country comparisons (e.g., Davidov et al., 2018). Yet, the lack of support for scalar invariance demands a degree of caution in interpreting the findings reported below. We think that the present research addresses an applied issue of significance and, given the relative absence of violence against doctors as a social issue in the U.S., these differences are unlikely to be entirely the result of response biases or other sources of error.

#### General Healthcare Uncertainty Scenarios

**Figure 1:**
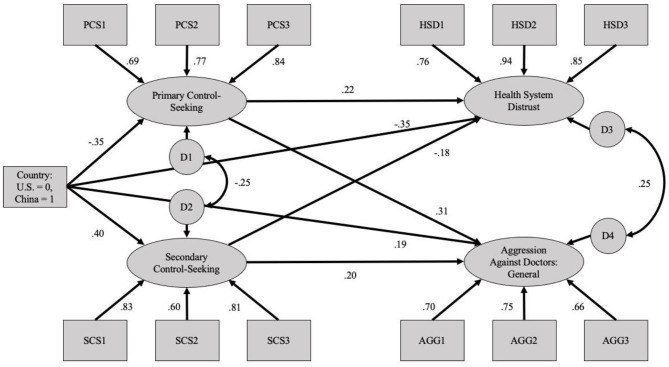
Full structural equation model using the general aggression against doctors scenarios (Study 2).

To assess the hypothesized mediation model, the data were fit to a structural equation model in which personal and external control were specified as mediators of national differences in the tendency to blame the health system vs. aggress against medical providers. In addition, given the likely relationship between the mediating (primary and secondary control-seeking) and outcome (health system distrust and aggression against doctors) variables, these pairs of factors were allowed to covary. Because the purpose of these analyses is to understand the relationship between the underlying latent factors, rather than the relationship between item-level, we applied a parceling method to increase model parsimony and improve the participant to parameter estimate ratio (Little et al., 2002). Thus, three parcels were calculated for shared decision-making, external locus of control, and health system distrust by randomly sorting and averaging items into three indicators per latent factor. The resultant model, along with factor loadings and standardized path weight estimates, is depicted in Figure 1. Though the Chi-square fit index was significant ($\chi_{\left(56\right)}^{2}$ = 458.95, *p* < 0.001), other fit indices that are less impacted by sample size suggest that the model's fit is within acceptable limits (CFI = 0.922; SRMR = 0.058; RMSEA = 0.088 [90% CI:0.081, 0.096]).

In addition to having acceptable fit, all of the latent factor loadings and path weights in the model depicted in Figure 1 were significant (*p* < 0.001). Generally, this model offers support for the present predictions, as Chinese participants (relative to Americans) reported greater levels of secondary control-seeking and aggression against doctors. In contrast, Americans (relative to Chinese participants) reported greater primary control-seeking and health system distrust. Further, the relationships between primary control-seeking and health system distrust on the one hand, and secondary control-seeking and aggression against doctors on the other hand, were both positive and significant.

**Figure 2:**
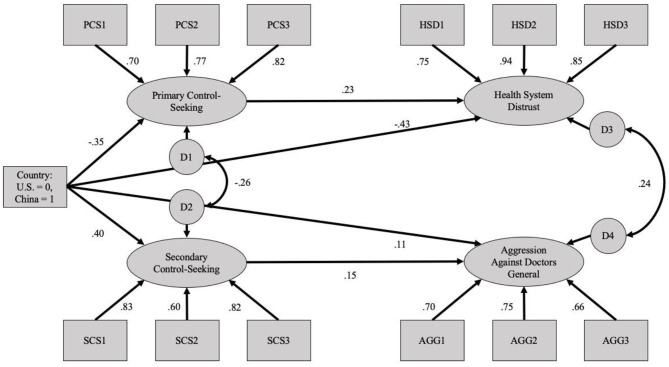
Mediation structural equation model using the general aggression against doctors scenarios (Study 2).

To more precisely test whether national differences in responses to medical uncertainty were mediated by the proposed constructs, a second model was examined in which cross-mediating pathway loadings (i.e., paths between primary control-seeking and aggression against doctors, and secondary control-seeking and health system distrust) were eliminated (see Figure 2). This model configuration allows for the examination of indirect effects through the hypothesized mediators by themselves. The mediation model also displayed acceptable, though less ideal, fit ($\chi_{\left(58\right)}^{2}$ = 530.44, *p* < 0.001; CFI = 0.908; SRMR = 0.078; RMSEA = 0.094 [90%CI:0.087, 0.102]). To examine the hypothesized mediating role of control preferences and to calculate bootstrap-based confidence intervals, the model was run with a bootstrapping approach utilizing 5,000 resamples. See Table 5 for indirect effects and confidence intervals.

**Table 5:** Analyses of indirect effects for the general healthcare uncertainty scenarios (Study 2).

**Path**	**Standardized indirect effect**	***p-*value**	**Unstandardized indirect effect**	**Unstandardized confidence interval**
Country → Primary control-seeking → Health system distrust	−0.081	<0.001	−0.129	−0.187, −0.081
Country → Secondary control-seeking → Aggression against doctors	0.059	0.013	0.078	0.025, 0.13

As indicated by the results reported in Table 5, the effects of country on both outcomes were partially mediated by the hypothesized constructs. In other words, while both of the direct relationships between country and health system distrust (*p* < 0.001) and aggression against doctors (*p* = 0.011) were significant, part of the national differences in these outcomes were accounted for by the proposed control-seeking preferences.

#### COVID-19 Specific Healthcare Scenarios

**Figure 3:**
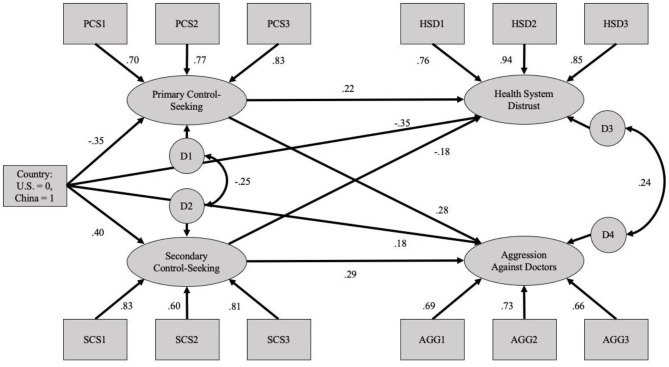
Full structural equation model using the COVID-19 uncertainty scenarios (Study 2).

**Figure 4:**
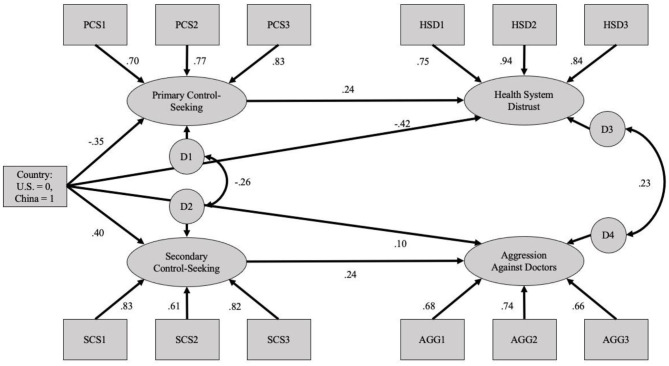
Mediation structural equation model using the COVID-19 Uncertainty Scenarios (Study 2).

Importantly for the present purposes, we also sought to determine whether the models could be replicated when considering the COVID-19 scenarios. Specifically, we examined the same models as above, but substituted the COVID-19-specific scenarios for the general uncertainty scenarios. The exact same analysis sequence was conducted, with a full path model being tested first (Figure 3), followed by a test that focused on the hypothesized mediating pathways (Figure 4). Analyses of the full model suggest an adequate fit to the data ($\chi_{\left(56\right)}^{2}$ = 485.97, *p* < 0.001; CFI = 0.916; SRMR = 0.058; RMSEA = 0.091 [90% CI:0.084, 0.099]), with all factor loadings and predicted paths yielding significant relationships (*p*s < 0.001).

Again, to explore the predicted mediational pathways more directly, we analyzed models in which the cross-mediating pathways were eliminated (Figure 4). This model again yielded adequate fit indices ($\chi_{\left(58\right)}^{2}$ = 547.86, *p* < 0.001; CFI = 0.905; SRMR = 0.077; RMSEA = 0.096 [90%CI:0.089, 0.103]). To assess the indirect relation between country and outcomes, through the hypothesized control-seeking mechanisms, we assessed those indirect effects with a bootstrapping method utilizing 5,000 resamples. The results of these analyses are depicted in Table 6.

**Table 6:** Analyses of indirect effects for the COVID-19 healthcare uncertainty scenarios (Study 2).

**Path**	**Standardized indirect effect**	***p-*value**	**Unstandardized indirect effect**	**Unstandardized confidence interval**
Country → Primary control-seeking → Health system distrust	−0.083	<0.001	−0.132	−0.192, −0.083
Country → Secondary control-seeking → Aggression against doctors	0.098	<0.001	0.146	0.094, 0.204

Once again, the confidence intervals for both indirect effects did not contain zero, suggesting that the national differences in health system distrust and violence against doctors (this time in COVID-19 scenarios) were partially mediated by the proposed control-seeking tendences.

#### COVID-Affected vs. Unaffected Areas and Positive Cognitive Reframing

To explore whether individuals' control-seeking and scapegoating tendencies were moderated by living in COVID-affected (vs. unaffected) areas, between-subjects ANOVAs were conducted in which the effects of nation, COVID-affected (vs. unaffected) area, and the interaction of these two factors were assessed on all measures included in the study. These analyses yielded non-significant main effects of COVID-affected area and country by area interactions (all *p*s > 0.05) for frustration and aggression in the general healthcare scenarios, frustration in the COVID-specific scenarios, primary control seeking, and health system distrust. There were, however, significant effects of living in a COVID-affected area for secondary control-seeking, positive cognitive reframing, and aggression toward doctors, though the latter main effect was qualified by a country by COVID-affected area interaction. See Table 7 for the full statistical results of ANOVAs that yielded significant results.

**Table 7:** ANOVAS of country, living in COVID-19-affected areas, and their interaction on secondary control-seeking, positive cognitive reframing, and aggression toward doctors in the COVID-19-specific scenarios (Study 2).

**Outcome**	**Predictor**	**df**	***F***	***p***	**Partial **η**^2^**
Secondary control-seeking	Overall model	3	55.45	<0.001	0.15
	Country	1	120.86	<0.001	0.12
	COVID area	1	11.09	0.001	0.01
	Country × Area	1	0.03	0.87	0.00
Positive cognitive reframing	Overall model	3	57.44	<0.001	0.16
	Country	1	119.63	<0.001	0.12
	COVID area	1	4.66	0.031	0.01
	Country × Area	1	0.30	0.59	0.00
Aggression toward doctors—COVID-19	Overall model	3	12.06	<0.001	0.04
	Country	1	34.26	<0.001	0.04
	COVID area	1	6.74	0.010	0.01
	Country × Area	1	4.43	0.036	0.01

**Figure 5:**
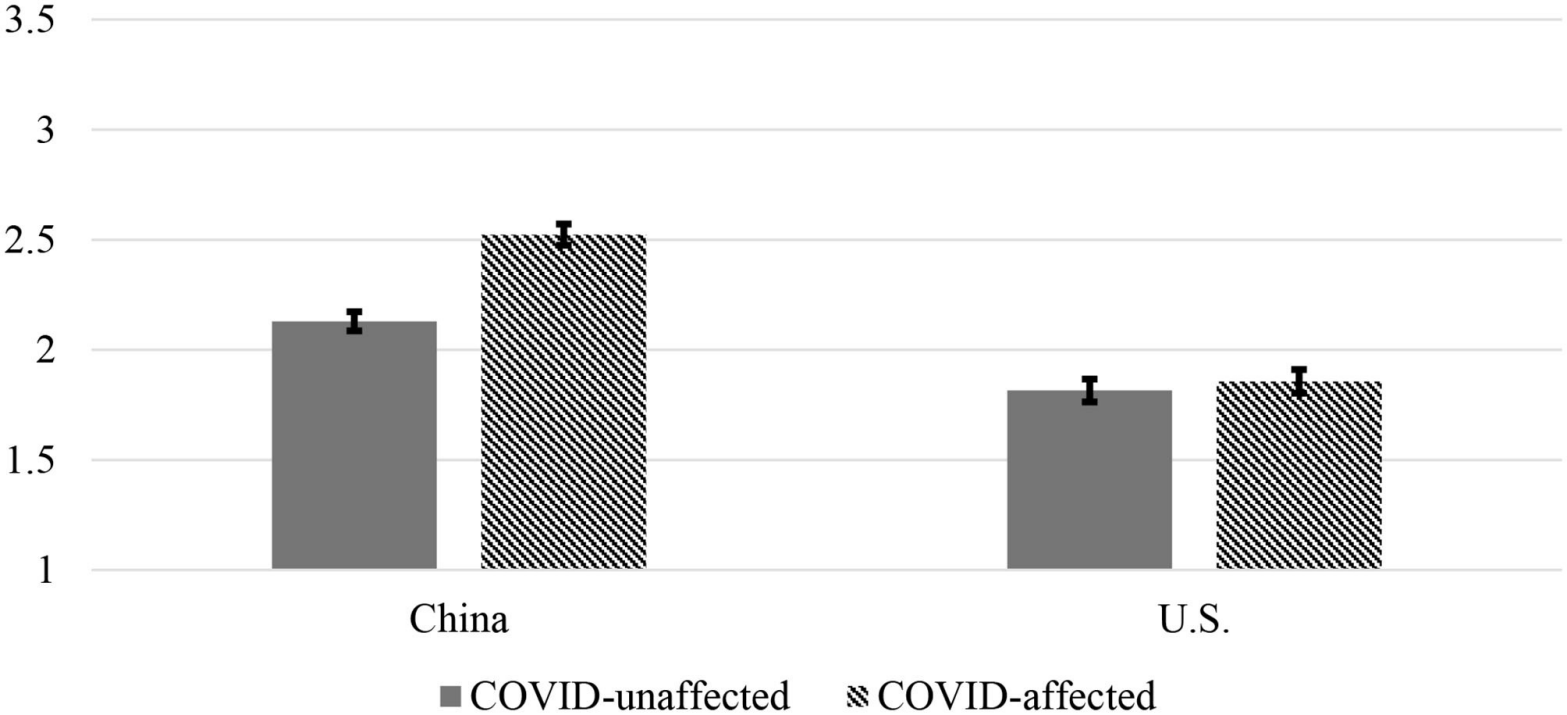
ANOVA results for country by COVID-affected area interaction on aggression against doctors (COVID-19 Scenarios) (Study 2). Error bars indicate standard errors.

The analyses depicted in Table 7 suggest that, in addition to national differences in most of the variables in Study 2 (see Table 3), whether or not participants lived in an area affected by COVID-19 was related to greater secondary control-seeking, positive cognitive reframing, and aggression toward doctors in the scenarios specific to COVID-19. This latter finding was qualified by a country by COVID-19-affected area interaction, such that the tendency for Chinese participants to want to aggress toward doctors (relative to American participants) was more extreme among Chinese living in COVID-19-affected areas (see Figure 5).

In terms of our exploratory variable of positive cognitive reframing, it was in fact the case that people in China engaged in this form of coping to a relatively greater extent. However, examination of mean levels of aggression against doctors in China between Studies 1 and 2 suggests that use of this coping mechanism did not dramatically mitigate the more negative defense mechanism of aggression.

### Discussion

A high-powered confirmatory study, Study 2 added several important pieces of information to the initial exploratory results obtained in Study 1. First, cross-cultural mean differences and cross-sectional patterns of association offered confirmatory support for our theoretical model. Replicating Study 1, Chinese (compared to U.S.) participants showed a relatively greater tendency to aggress against doctors in hypothetical scenarios involving both general medical uncertainty and COVID-19. Also replicating Study 1, U.S. (compared to Chinese) participants showed higher levels of distrust in the health system. Importantly, extending on Study 1's initial findings, we also found support for our multiple mediation model, such that the cross-cultural differences in outcomes were partly mediated by variation in control-seeking. U.S. (compared to Chinese) participants seek primary control to a greater extent, which is related to their relative tendency toward health system distrust; and Chinese (compared to U.S.) participants seek secondary control to a greater extent, which is related to their relative tendency toward aggression against doctors.

Importantly, this model replicated (for aggression against doctors) in both the context of general medical uncertainty, and COVID-19 specific, scenarios. Relevant to the current necessity for understanding how people respond to global pandemics, there were interesting patterns related to COVID-19 in the data, some of which appeared culturally generalizable, and one that was culture-specific. In particular, in both countries, reporting living in an area that was severely impacted by COVID-19 was associated with secondary control strategies, in particular more secondary control-seeking in the medical context (Powerful Others HLOC) as well as positive cognitive reframing. Finally, and attesting to the importance of our scapegoating conceptualization, we found that the cross-cultural difference in tendencies to aggress against doctors (in the COVID-19 scenarios) was moderated by living in a COVID-impacted environment, such that, among Chinese participants, greater tendencies to aggress were observed among participants living in more impacted areas.

## General Discussion

Distrust and discord between patients, physicians, and the healthcare system is a major and growing international problem. The present paper applies a novel explanation for this phenomenon drawing on a conceptualization of cultural pathways to scapegoating in the face of medical uncertainty. It draws on prior work addressing the specific issue of violence against doctors in China from a scapegoating perspective (Yang et al., under review) to propose and test a theory of how Chinese and U.S. culture afford different viable scapegoating targets in the health domain, in order to satisfy varying needs for primary and secondary control. This work therefore importantly extends our understanding of the psychology of control and trust to a prominent applied context, one that has more relevance than ever before in light of the massive health-related uncertainty caused by the COVID-19 pandemic.

From one vantage point, our findings speak to processes that generalize across cultures, even if they manifest in slightly different ways (Kitayama et al., 2010). People living in both China and the United States tend to scapegoat certain viable targets when encountering medical uncertainty for themselves or their relatives. It is significant that our confirmatory Study 2—conducted under conditions of a global pandemic—yielded essentially similar support for these general tendencies as was observed in Study 1 (pre-pandemic), suggesting a degree of both cross-cultural and historical stability.

On the other hand, we observe consistent cultural variation in the specific manifestation of scapegoating tendencies in the face of medical uncertainty, as well as the processes driving these tendencies. Replicating prior research on scapegoating (Yang et al., under review) as well as the cultural psychology of trust (Zhang et al., 2019), people in China (vs. the United States) had a greater tendency to aggress against local healthcare workers in situations of medical uncertainty. By contrast, people in the United States (vs. China) showed relative tendencies to distrust the healthcare system as a whole. Further, these culture-level differences in scapegoating mechanisms were partially mediated by different patterns of control-seeking.

The observed cultural differences in primary and secondary control-seeking are consistent with previous findings. Historic conditions favorable to individualism have given rise to strong motives for primary personal control in the United States, but people in China and other Asian cultures have historically favored patterns of acceptance and adjustment to the status quo (Kay and Sullivan, 2013). At the same time, the state of illness itself forces upon the patient a strong sense of uncertainty and lack of control. The COVID-19 pandemic in particular has posed a strong threat to people's sense of control in many settings around the world; but just as socio-political, public health, and economic responses to the crisis have varied as a function of cultural context, so too will the psychological defenses people employ against the threat to control posed by this tidal wave of medical uncertainty.

### Limitations

Given that this research stemmed from prior applied work on the phenomenon of violence against doctors in China (Yang et al., under review), and additionally sought to examine a second important applied phenomenon—healthcare system distrust in the COVID-19 context—we approached study design from a more applied perspective. In other words, we prioritized operationalizing our theoretical constructs in ways that were highly germane to the context of healthcare and the doctor-patient relationship, as well as not including additional, more abstract measures in order to avoid participant fatigue. This was particularly the case for our confirmatory Study 2 design. These decisions came at a cost to the theoretical clarity of our data. For example, although we used a scapegoating framework to develop our hypotheses, we did not directly measure attributions of blame in the current studies, an important component of scapegoating that we have in fact measured in earlier studies of aggression against doctors (Yang et al., under review). And although there are more direct measures of primary and secondary control available (e.g., Heckhausen et al., 1998), we elected instead to use measures specifically intended for the way these processes manifest in the healthcare domain, e.g., in terms of vicarious control-seeking in the doctor-patient relationship. Ultimately, these decisions limited our ability to definitively test our theoretical framework in this applied context. Nevertheless, given that the patterns of data support our hypotheses, and that we developed these hypotheses from an underlying framework, the findings are at least consistent with a theory of cultural pathways to scapegoating.

Some researchers might also consider the fact that we selected measures for inclusion in our confirmatory Study 2 based partly on their performance in our exploratory Study 1 to be another limitation of the present research. From this perspective, it could be argued that we selected the measures that were most likely to support our theoretical account, while ignoring relevant measures that might have cast doubt on the framework. While we concede that some researchers may view our approach in this light, we personally feel that this represents a confusion between exploratory data analysis and what are referred to as “questionable research practices” (Jebb et al., 2017). Because we have openly acknowledged that Study 1 was conducted in an exploratory spirit, any conclusions from that study need to be interpreted with due caution. However, the aim of exploratory data analysis is often to develop theory and methods for future confirmatory study (Jebb et al., 2017), which is exactly the approach we adopted here. We did not focus on new or specific measures of primary and secondary control-seeking in Study 2 simply because they “performed” in Study 1, but also because the patterns were consistent with prior research and our theoretical account. For instance, in hindsight, the choice to operationalize primary and secondary control in Study 1 using measures of *presence* rather than *desire for* control was a poor design choice based on our theoretical framework. Accordingly, we selected different measures for inclusion in Study 2, and these data provided confirmatory evidence for our account.

Nevertheless, it is important for future research to attempt to further replicate the pattern of results seen in these studies, which remain applied and somewhat preliminary in nature. Beyond the outcome variables, our studies also attest to the ongoing need for further examination of the relationship between *need for* and *presence of* primary and secondary control. Ideally, future work would investigate these phenomena from a more purely theory-driven perspective; as stated, the applied nature of our work in the healthcare context limited our ability for theory refinement.

### Practical Implications

The concept of “uncertainty in illness” (Mishel, 1988) explains the patient's treatment of disease-related stimuli. Patients often (1) do not know the precise symptoms of the disease; (2) do not understand the generally complicated methods of treatment and care; (3) lack information related to the diagnosis and severity of the disease; and (4) recognize that the course and prognosis of the disease cannot be predicted with certainty (Mishel, 1988; Maikranz et al., 2007). The COVID-19 pandemic has exacerbated these processes of uncertainty in illness for many people, given the highly contagious nature of the disease, its disproportionate impact on certain vulnerable individuals, and a general lack of certainty about the disease among health professionals, particularly in the early days of the pandemic (Rettie and Daniels, 2020). Within this general context of uncertainty in illness, it is important to consider the nature of the doctor-patient relationship. The patient is at a disadvantage when it comes to information and resources (Goodyear-Smith and Buetow, 2001). Being ill results in a sense of uncontrollability focused on the possible future threat, danger, or other upcoming, potentially harmful events (Beisecker, 1990).

According to our framework and present pattern of results, Chinese individuals are motivated to adopt secondary control strategies to compensate for lack of personal control attendant on the experience of illness. Perhaps unsurprisingly, because Chinese individuals wish to place their faith in powerful others (healthcare workers) to control and resolve their illness experience, they resolve continued frustrations and uncertainties by blaming, and even aggressing against, these local representatives of the healthcare system. In comparison, U.S. residents seem motivated to maintain a sense of primary control despite the inherent uncertainties of the illness experience. However, in this cultural context of trust, aggression against doctors is not an afforded response; rather, those seeking greater primary control blame the broader healthcare system for their negative illness experiences. This attributional style may allow these individuals to maintain the perception that they can locally control their health (e.g., through lifestyle choices or asserting agency in the doctor-patient relationship), at the same time that they trace their health problems to broader systemic factors.

While the current research has focused on investigating problematic tendencies (i.e., scapegoating motivations) within the two cultural settings, this comparative research also highlights the fact that national leaders and healthcare professionals stand to learn from each other by recognizing divergent cultural strengths. For instance, the Chinese government has continued political support for its healthcare reform from 2009 until now, enabling conditions to achieve national universal health coverage (Tao et al., 2020). The health insurance system has been reformed and different kinds of medical insurance have combined to promote health equity (Meng et al., 2015). It is possible that these recent efforts on the part of the Chinese government contribute to laypeople's relative trust in the healthcare system as a whole. Given the calamity posed by COVID-19 in the United States, and the role that was likely played by distrust in the healthcare system, it is important to recognize the potentially pernicious consequences of this distrust. At the same time, in the United States people seem to maintain a general respect for the healthcare professions, and tend to respect and trust their individual doctors even if they devalue the healthcare system as a whole (Hall, 2005). Given the ongoing dilemma of violence against doctors in China, social leaders and public health professionals might look to the structure of doctor-patient relationships in the United States for insight into how to restore a sense of trust between individual patients and their local providers.

Generally speaking, our data underscore the importance of considering unique cultural pathways to trust and scapegoating in the context of medical uncertainty, especially when it comes to the important questions of what local practitioners and state/federal policymakers can do to improve trust and decrease scapegoating. For instance, in the United States, relative levels of trust in and aggression against local practitioners is not the most pressing issue; instead, trust in the healthcare system as a whole needs to be addressed. This suggests the importance of policy, regulation, transparency, and clear communication regarding issues such as insurance, pharmaceuticals, and vaccines at the broader federal level in the United States. However, the opposite pattern in China may prevail, which suggests that local healthcare workers may be well-advised to pursue individual-level solutions to establish and maintain patient trust (see Wolfensberger and Wrigley, 2019). In both cultures, however, our data also point to the importance of meeting patient needs for control in this context, in whatever manner those needs may be culturally shaped.

## Data Availability Statement

The raw data supporting the conclusions of this article will be made available by the authors, without undue reservation.

## Ethics Statement

The studies involving human participants were reviewed and approved by University of Arizona IRB. The patients/participants provided their written informed consent to participate in this study.

## Author Contributions

QY and DS designed the studies and facilitated data collection. IY performed primary data analysis. JW facilitated data collection and completion of the studies. QY, DS, and IY drafted the manuscript. All authors approved revisions and final version of the manuscript.

## Conflict of Interest

The authors declare that the research was conducted in the absence of any commercial or financial relationships that could be construed as a potential conflict of interest.

## Footnotes

^1^Of course, we do not argue that scapegoating is the only, or even the most prominent, defensive psychological response to medical uncertainty. But given our interest in addressing the important applied phenomena of aggression and distrust against healthcare workers and the healthcare system, it is probably one of the most important responses to understand, and hence the focus of our empirical efforts. It is also important to acknowledge that scapegoating can have many important motivations and consequences (e.g., Rothschild et al., 2012), but we focused in the present context on its control maintenance function.

^2^Study 1 also included a measure of belief that medicines are overprescribed by doctors, taken from the larger Beliefs about Medicine Questionnaire (Horne et al., 1999). We included this measure because in media reports about violence against doctors in China, a common complaint voiced by members of the public is over-prescription of expensive medicines. Results for this measure indeed indicated that, among Chinese participants, belief about overuse of medicine is significantly associated with aggression against doctors. However, because this is a culture-specific effect independent of our broader theoretical, cross-cultural model, we did not include this measure in Study 2, and do not focus on the results from this measure in our reporting of Study 1.

^3^While gender was not a focus of this investigation, we conducted additional analyses in which we controlled for gender in order to rule out the possibility that the uneven gender representation could be driving nation-level differences. Controlling for gender did not affect any of the nation-level differences reported below. Further, the only variables that displayed main effects for gender were Internal Health LOC [*t*_(642)_ = 3.55, *p* < 0.001, *d* = 0.79] and frustration at the healthcare scenarios [*t*_(642)_ = −3.26, *p* < 0.001, *d* = 0.96], such that males scored higher than females on Internal Health LOC and females reported more frustration than males.

^4^Because the gender distribution between the U.S. and China was not even, we again examined whether all the nation-level differences reported below persist when controlling for gender. Controlling for gender did not eliminate any of the effects reported below. Further, main effects of gender were only observed for general aggression toward doctors [*t*_(914)_ = 2.70, *p* = 0.007, *d* = 0.78] and COVID aggression toward doctors [*t*_(914)_ = 2.52, *p* = 0.012, *d* = 1.05], such that males reported greater desires to aggress in both sets of scenarios. Because gender differences were not a focal point of this research, we do not further report further analyses of gender.
